# Thermal, Physico-Chemical, and Mechanical Behaviour of Mass Concrete with Hybrid Blends of Bentonite and Fly Ash

**DOI:** 10.3390/ma12010060

**Published:** 2018-12-25

**Authors:** Muhammad Zeeshan Ahad, Muhammad Ashraf, Rabinder Kumar, Mukhtar Ullah

**Affiliations:** 1 Department of Civil Engineering, Iqra National University, Peshawar 25000, Pakistan; 2 Department of Civil Engineering, GIK Institute of Engineering Sciences and Technology, Topi 23460, Swabi, KPK, Pakistan; matanoli@giki.edu.pk (M.A.); rabinder.kumar@giki.edu.pk (R.K.); 3 Department of Electrical Engineering, FAST National University of Computer and Emerging Sciences, Islamabad 44000, Pakistan; mukhtar.ullah@nu.edu.pk

**Keywords:** mass concrete, thermal analysis, TGA/DTA, XRD, mechanical properties

## Abstract

Mass concrete has been commonly known for its thermal stresses which arise due to the entrapment of hydration temperature susceptible to thermal cracking. The utilization of mineral additives is a promising and widely adopted technique to mitigate such effects. This paper presents the thermal, physico-chemical, mechanical, and morphological behaviour of mass concrete with blends of bentonite (BT) and fly ash (FA). Apart from the rise in temperature due to hydration, the compressive strength, ultrasonic pulse velocity (UPV), differential thermal analysis (DTA), thermo-gravimetric analysis (TGA), X-ray diffraction (XRD) analysis, and microstructure were studied. The results of this study revealed that the substitution of BT and FA significantly improved the compressive strength and development rate of UPV in the mass concrete samples. The FA concrete (FC) specimen presented the lowest temperature during the peak hours compared to all other concrete mixes studied in this research. Bentonite concrete (BC) was also found to be more effective in controlling the escalation of temperature in mass concrete. Scan electron microscopy (SEM) micrographs presented partially reacted FA particles in a mix. XRD and DTA analysis indicated that the concentration of calcium hydroxide (CH) declined by substituting FA and BT, specifically in ternary blends, which was due to the dilution effect and consumption of CH through the pozzolanic reaction.

## 1. Introduction 

Thermal stresses in mass concrete often rise due to the hydration of cement. The temperature differences in mass concrete may vary during its placement [1,2]. The temperature at the core is significantly higher than at the surface due to the entrapment of heat that adversely affects the concrete properties owing to thermal stresses and may lead to cracking [3]. To ensure the durability and quality of concrete used in complex construction projects, it is essential to observe and control the concrete mix temperature, i.e., ambient temperature and differential temperature in mass concrete elements. The structural integrity of concrete elements may be compromised due to the formation of thermal cracks [2,3,4]. This causes the exposure of reinforced concrete structures to chlorides, sulphates, and other aggressive agents which affect the long-term durability and serviceability of concrete structures [5].

Various methods are adopted to control the temperature in mass concrete based on their availability, handling, and cost effectiveness. The most common way to reduce the thermal effect in concrete is to utilize pozzolanic materials by substituting the bulk proportion of cement in mass concrete production. The decrease in the amount of heat of hydration and thermal stresses is related to the decrease in the amount of cement [6]. The pozzolanic materials are siliceous or alumina-siliceous materials, which chemically react with calcium hydro-oxide (CH) in the presence of moisture at an ordinary temperature to produce a mix with cementitious characteristics [7,8]. The most commonly used pozzolans in the production of concrete are fly ash (FA); silica fume (SF); metakaoiln (MK); and ashes obtained from agro-waste materials, such as rice husk, bagasse, and palm kernel shells [9,10]. Generally, the pozzolanic materials are used within the range of 20% to 30% as a cement replacement material (CRM) by weight of cement. Some studies also report the use of CRM in concrete beyond 30% [11]. FA is the most common material used in the concrete to retard the hydration of OPC at an early age. It has also been utilized as a replacement of cement in catering environmental deterioration due to the emission of CO_2_ during the manufacturing process of cement [12]. The estimated range of heat contribution of FA at an early age varies from 15% to 35% of the heat contribution from a similar amount of cement [13]. The total heat of hydration of FA mainly depends on the lime (CaO) content. The heat of hydration in FA concrete is controlled by various factors, i.e., quantity and chemical composition of cement and fly ash in a mix, ambient temperature, finesse of cement, and FA [14]. The replacement of FA with cement generally declines the heat development due to the slower hydration reaction. 

Bentonite (BT) is an alumina-siliceous material that produces a high amount of aluminium and siliceous compounds during its pozzolanic reaction. Pakistan has large and abundant reservoirs of BT clay, which is used for several industrial applications. However, its applications in the construction sector are not well-documented among all the well-established pozzolanic materials, such as FA, SF, rice husk ash (RHA), and MK, etc. There are few studies which report the effects of BT on concrete mechanical and durability properties. Memon et al. [15] studied the effects of BT on the workability, compressive strength, water absorption, and acid attack resistance of concrete and reported that a higher replacement of BT led to a reduction in the workability. In contrast, a higher strength was observed at 28 days and 56 days. In addition, BT mixes also performed better in acid attack resistance. In contrary, Ahmad et al. [16] found a reduction in the compressive strength with a higher substitution of BT in concrete mix. A similar trend was also reported by Akbar et al. [17]. Substation of 30% BT as cement replacement showed a reduction in the water absorption of concrete [16]. Mesboua et al. [18] reported a reduction in the workability and compressive strength in BT-cement grout. 

Several researchers have examined the effects of BT on the mechanical and durability properties of normal concrete. However, its effects on physico-chemical, thermal, and morphological properties of mass concrete have not been well-explored. Therefore, this study was designed to assess the effectiveness of BT as partial replacement to the cement on the aforementioned properties of mass concrete. Since the FA is the most commonly used pozzolan in concrete, mass concrete mixes containing FA separately and together with BT were also prepared to compare the performance of BT with FA and control mix.

## 2. Materials 

All materials were characterized for their chemical composition by the X-ray florescence (XRF, Axios FAST, Malvern Panalytical, Royston, UK) technique prior to their use in concrete. Type I ordinary Portland cement (OPC) confirming the requirements suggested by ASTM C150 [19] was utilized throughout the experimental program. Crushed aggregates with a maximum size of 20 mm and specific gravity of 2.67 were procured from a local quarry. Class F FA was used, after confirming the requirements suggested by ASTM C618 [20]. The processed commercial BT was utilized in concrete mixes with a specific gravity of 2.21. The chemical compositions of OPC, FA, and BT are provided in Table 1.

## 3. Experimental Program 

The experiment program was carried out to investigate the physico-chemical, mechanical properties, and thermal effect on binary and ternary blended concrete mix prepared with FA, BT, and OPC. Four different types of concrete samples were prepared with different proportions of FA and BT as substitutions to OPC by mass in concrete mix, as presented in Table 2. The mix was designed for a compressive strength of 25 ± 2 MPa. All mixes were prepared with a constant water to binder ratio of 0.5. Details of the mix proportion are presented in Table 3. The acronyms for concrete mixes were adopted in such a way that they clearly signify the type of pozzolan used in a concrete mix. The mixes were designated as control concrete (CC) without any pozzolanic material, FA concrete (FC), BT concrete (BC), and a combination of FA and BT concrete (FBC). 

**Figure 1:**
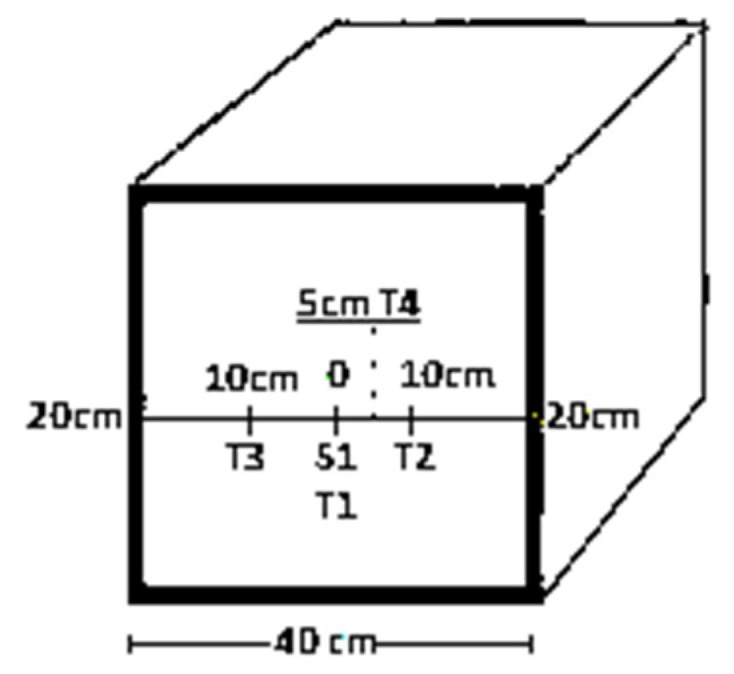
Location of thermocouples in a mass concrete specimen.

In total, 15 standard sized cylinders (15.24 cm × 30.48 cm) and four massive concrete blocks with dimensions (60 cm × 60 cm × 40 cm) were cast, i.e., control concrete (CC), 30% FA (FC), 30% BT (BC), and a combination of 15% FA and 15% BT (FBC), respectively. The fresh concrete was poured in layers and compacted using vibrators (WT-C0547-0216, Wisdom, Chongqing, China) to remove air voids. Four embedded thermocouple sensors (MAX6675 K Type, Shenzhen, China) were placed in the central and outer parts of mass concrete blocks to record the temperature, as shown in Figure 1. The blocks were then covered with wet coverings for the purpose of curing till 91 days. 

**Figure 2:**
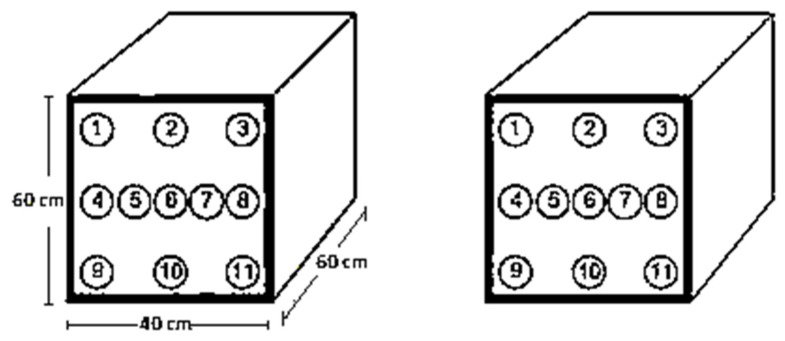
Core extraction pattern from massive concrete blocks.

The experiments were performed in two phases. In the first phase, an increase in the temperature was recorded after the casting of the concrete block. The temperature deviation was declined after reaching its peak values. The test was terminated when the declined temperature in the central zone attained a similar temperature value to the surface zone in the mass concrete block. In the second phase, besides the compressive strength test, the ultrasonic pulse velocity (UPV, Ultrasonic Tester C-1920A, Elmsford, NY, USA) was measured at 7, 14, 28, 56, and 91 days of curing. The compressive strength behaviour and UPV values were also recorded on mass concrete blocks. The blocks were divided into two parts, named surface (outer) and central (core), as shown in Figure 2. In total, 21 cores were taken out from the outer and the central part of concrete blocks for the testing of compressive strength and UPV after 91 days of curing. The difference in temperature and loss of mass with respect to temperature in concrete specimens containing FA and BT were analysed through the Differential thermal analysis (DTA, PerkinElmer Diamond Series, Waltham, MA, USA) and Thermo-gravimetric analysis (TGA, PerkinElmer Diamond Series) technique at 28 days. In addition, the X-ray diffraction (XRD, JDX-3532, Jeol, Tokyo, Japan) technique was used to analyse the mineralogical composition of concrete samples. The microstate of samples was examined through the scanning electron microscopic (SEM, JSM-5910, Jeol) technique at 28 days of curing.

## 4. Results and Discussion 

### 4.1. Compressive Strength for Cylinders

**Figure 3:**
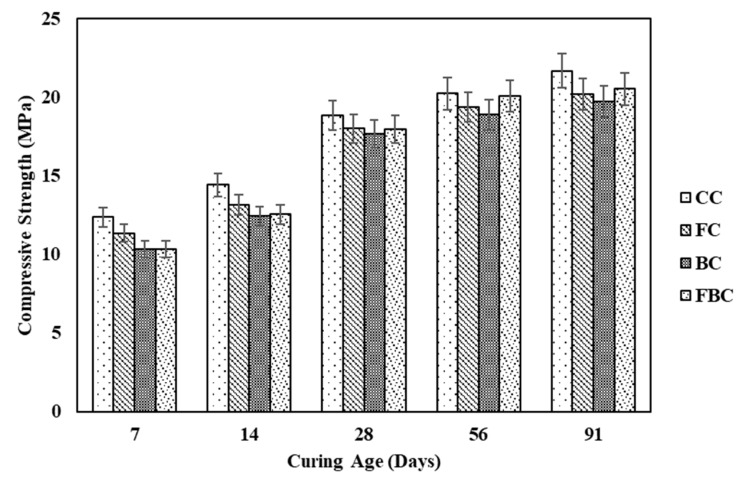
Compressive strength of mass concrete samples containing FA and BT.

This property of concrete is considered a basic design parameter of concrete structures. All other properties of concrete can be judged on the basis of compressive strength. Figure 3 shows the individual and combined behaviour of BT and FA in compressive strength concrete specimens. The outcomes from the compressive strength test exhibited that the strength development of all the specimens was directly associated with the curing age. Overall, the grade of concrete with respect to its design strength at 28 days was approximately similar. The inclusion of FA and BT in concrete presented a slight reduction of 4% and 6% in compressive strength, respectively, at 28 days. A similar trend was continued up to the curing age of 91 days. The decrease in strength is mainly attributed to the reduction in the amount of cement content, which resulted in a slow pozzolanic reaction [21]. The ternary blending of FA and BT presented a reduction in the magnitude of strength at an early age, but at 28 days, identical values of FC and FBC were obtained. At 56 days of curing, the strength was approximately similar in comparison to the control concrete. However, the strength development rate was decreased at 91 days. 

**Figure 4:**
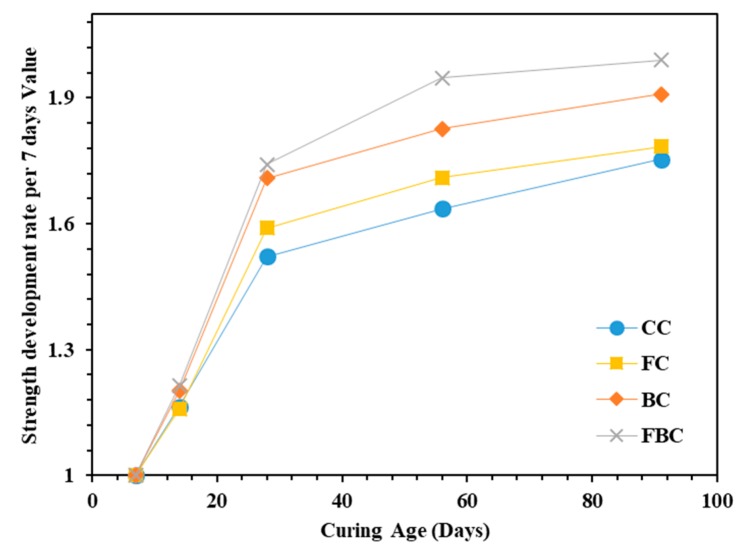
Strength development rate for mass concrete samples containing FA and BT.

The rate of strength development was also determined with reference to the value of compressive strength obtained at seven days [22,23] (Figure 4). The values showed that the development rate of compressive strength was improved with the substitution of FA and BT in FC and BC compared to CC. The strength development in FC is attributed to the fineness, proportions, and type of FA [24]. In the ternary blended mixture, FBC presented a greater strength development rate at all ages than other concrete mixes studied in this research. This can be attributed to the inclusion of BT, which acted as an activation agent to increase the strength development rate. The similar increment rate of the compressive strength was observed in CC and FC specimens till 14 days. However, the increment rate was gradually increased for FC at later ages up to 91 days. This can be attributed to the higher pozzolanic activity (i.e., formation of additional calcium silicate hydrate) due to the presence of FA during the pozzolanic reaction [25]. 

### 4.2. Ultrasonic Pulse Velocity (UPV) for Cylinder Specimen

**Figure 5:**
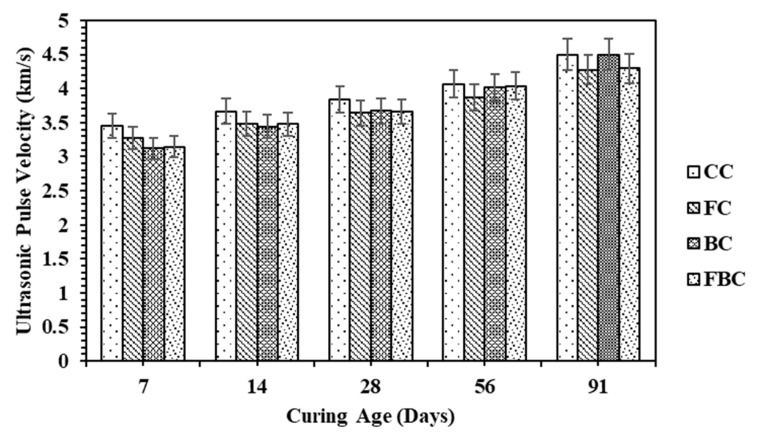
UPV values for concrete cylinders containing FA and BT.

Ultrasonic pulse velocity (UPV) is a non-destructive test used to quantify the strength of concrete. UPV results are affected by the density and internal cracks of a concrete specimen. The data for UPV values are presented in terms of km/s. This test was performed on cylinder specimens and mass concrete specimens. The values for UPV on cylinder specimens containing mineral additives at 7, 14, 28, 56, and 91 days are shown in Figure 5.

At an early age, FC showed a lower value compared to CC, but a value higher than that of BM and FBC. It was then gradually increased with respect to time. In addition, BC also presented lower values than CC till 56 days, and the value was closer to CC at 91 days. The reduction in the values were due to the lower hydration. The hydration rate in normal concrete was high compared to the concrete containing pozzolanic materials. It is observed that BT has a good pozzolanic reactivity since the values continued to increase until 91 days. The significant increments in UPV value for CC were attributed to the denser microstructure. The FBC showed almost the same pattern as FC. However, slightly smaller values were recorded at initial ages, and thereafter, an elevated curve was observed at 56 days.

**Figure 6:**
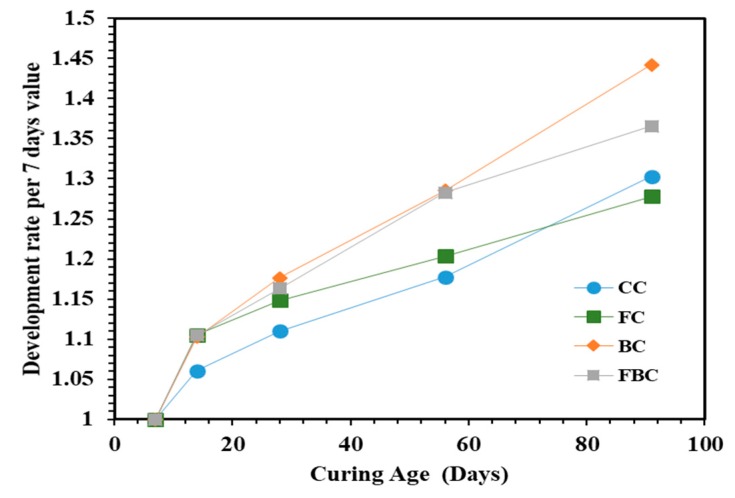
Development rate of UPV values for concrete cylinders containing FA and BT.

Based on UPV values, the concrete’s quality was characterized by the criteria presented in Table 4. In this research, the UPV values were noted between 3.1 to 4.5 km s^−1^ for all cylinder specimens at all curing ages, which indicated that the type of concrete was in the range of “good concrete”. The influence of BT and FA on the development rate of UPV of cylindrical concrete specimens is shown in Figure 6. 

The rate of development in UPV values was observed to be lower at early ages, but it gradually increased with respect to time. The specimen containing FA and BT presented a more significant development rate in UPV values than CC. The substitution of FC showed the same development pattern as CC, but a significant reduction was observed at 91 days. The BC showed continual development with respect to concrete age, which shows that the UPV values of these specimens progressively improved with time.

Ternary blends of BT and FA presented a similar magnitude of UPV values at 56 days of curing. However, it was found to be higher in BC at later ages. At 91 days, the rate of development of samples was observed in the following order: BC > FBC > CC > FC. Generally, the value of UPV is influenced by the microstructure of concrete. The higher development rate in BC can be attributed to its improved microstructure. The fine particles of pozzolans eliminated the capillary pores by forming additional silicates during the pozzolanic reaction process.

### 4.3. Compressive Strength and UPV for Cores

At 91 days, the cores of 101.6mm φ were taken out from mass concrete blocks. The core extraction pattern is shown in Figure 2. The extraction of cores was performed across a 600 mm dimension from the front and back plane of the mass concrete block and signified as “outer cores”, and those extracted from the centre were identified as “Inner cores”. The comparison between the compressive strength and UPV values of inner and outer cores is demonstrated in Table 5. 

**Figure 7:**
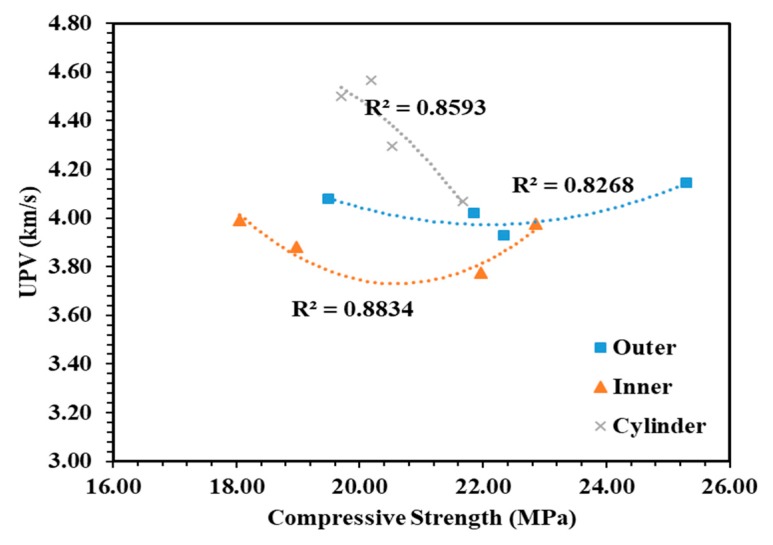
Relationship between compressive strength and UPV values of inner cores, outer cores, and cylinder specimens of all four groups at 91 days.

A total of 12 surface cores and 10 central cores were taken out from each massive concrete block, having dimensions similar to the size of cylinders [27]. The average value of UPV was obtained by testing two core specimens from each region. It can be seen from Table 5 that the lowest UPV values were obtained for central cores of each mass concrete specimen. Similarly, from the data of the core strength test, it can be clearly seen that inner cores have less strength development compared to the outer core exposed to the environment. Moreover, a closer reading was obtained between the cores extracted from regions exposed to the outer environment and cylinder specimens. The main reason for this is due to the higher temperature at the cores, where the heat was trapped, which caused a significant increase in the rate of cement hydration. Eventually, the concrete was subjected to cracks and lowered the strength and UPV values. The relationships between compressive strength and UPV values of inner cores, outer cores, and cylinder specimens are presented in Figure 7. Based on the responses, the significant correlation between compressive strength and UPV was obtained with an R^2^ coefficient of 0.826, 0.883, and 0.859 for inner cores, outer cores, and cylinder specimens, respectively. This showed a significantly strong relationship between the parameters. Less variation in UPV values was observed between the surface and central cores containing FA and BT. Hence, the binary and ternary blends of FA and BT presented the homogeneity of mass concrete specimens. 

### 4.4. Changes in Temperature

**Figure 8:**
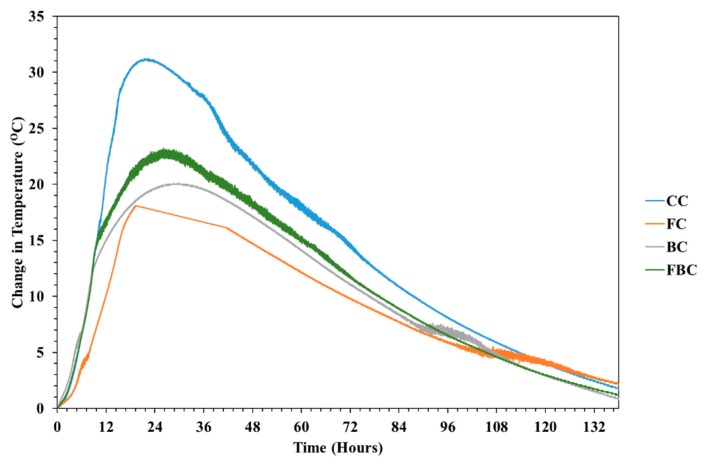
Temperature variations with respect to time on mass concrete specimens.

The result obtained from variation in the temperature at the core of mass concrete samples is presented in Figure 8. It can be seen that CC presented a steep rise in temperature, resulting in a maximum temperature of 32.5 °C compared to the other mass concrete mixes containing mineral additives (BT and FA). A significant reduction in the temperature was observed with the addition of FA and BT separately and in combination. The substitution of FA and BT in concrete mixes (FC, BC, and FBC) resulted in the decrease of 44%, 40%, and 29%, respectively. It was observed that the evolution of heat was higher at early ages and cement hydration was initiated after final setting in all concrete samples.

The rate of temperature rise in FC was much slower than CC. The peak temperature was recorded as 18.8 °C after 19 h of casting. The rate of rise in the FBC was slower than CC, but compared to BC and FC, was comparatively higher. The peak temperature was recorded as 23.3 °C after 25 h of casting. The use of BT (BC) with 30% partial replacement of ordinary Portland cement showed a reduction in peak temperatures of 33%, whereas using fly-ash (FC) with similar replacement of ordinary Portland cement showed a reduction in peak temperature of 40.8%. The combination of Fly-ash BT (FBC) replacement showed a reduction of the peak of 27%. 

It has been reported that the substitution of 50% FA can lower the concrete peak temperature by 23%, whereas the individual substitution of FA at a moderate level (20–30%) may not contribute to significantly lowering the heat evolution in concrete [28]. In this research, the substitution of FA separately presented a significant reduction in the peak temperature, which can be attributed to the quality of FA. The possibility of external and internal cracks in concrete increases with more than a 20 °C difference in temperature [29]. The difference in the surface and core temperature of CC was recorded as 37.7 °C, whereas in the cases of BC, FC, and FBC, the differences in surface and core temperature were 20.1 °C, 25.9 °C, and 29.2 °C, respectively. The effect of BC, FC, and FBC was apparent in lowering the temperature, as noticed from the results obtained.

### 4.5. Thermal Analysis (TGA/DTA)

**Figure 9:**
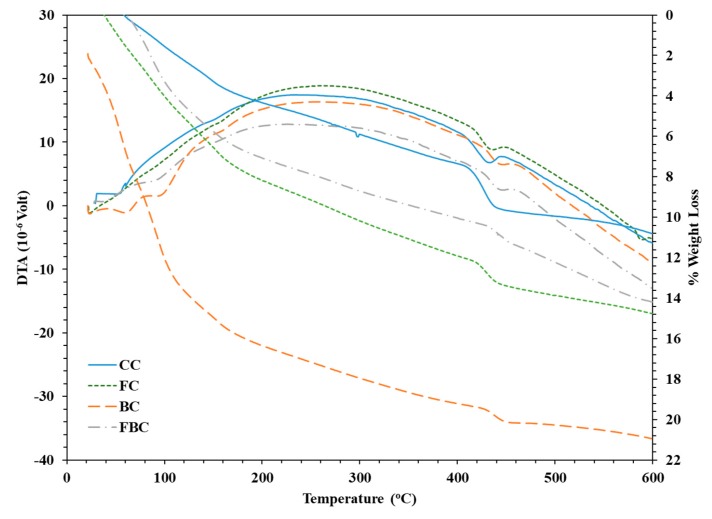
TGA/DTA of mass concrete specimens containing mineral additives at 28 days.

Differential thermal analysis (DTA) is a technique that observes the variation in the temperature of the specimen with respect to temperature or time. Generally, a difference in temperature is indicated by electric potential (µV). The Thermo-gravimetric (TGA) technique is used to measure the reduction in mass of a specimen with respect to temperature. The thermal decomposition of different phases in a material is identified through DTA analysis, whereas loss in weight due to decomposition is simultaneously measured thorough TGA. The TGA/DTA thermo-grams of CC, FC, BC, and FBC samples were analysed at the age of 28 days (Figure 9). The identification of calcium silicate hydrates’ (C–S–H) endo-thermal peak was observed within the temperature range of 115–225 °C, whereas calcium hydroxide (CH) and calcium carbonates (CaCO_3_) have been observed in the range of 430–550 °C and 550–650 °C, respectively [22]. 

The calcium hydroxide was consumed at a higher proportion across all specimens containing BT and FA at 28 days, which represents the formation of an increased amount of C–S–H due to the pozzolanic reaction. The addition of FA as a replacement for OPC in FC presented a hump at 550–650 °C, which shows the presence of crystalline silica. The loss in the mass due to the temperature of specimens containing BT and FA is presented in Figure 9. A loss in the mass was observed at 420–470 °C, which could be attributed to the decomposition of CH. The decline in the curve beyond 470 °C was due to the final stage of dehydration of C–S–H. The loss of weight between 0 and 150 °C signifies the loss in the moisture in the tested samples. 

The reduction in CH weight of all specimens containing FA and BT presented lower values than CC. The percentage loss was calculated with these values against percentage loss in CC. The drop of CH amount in FC, BC, and FBC was calculated as 33%, 40%, and 28%, respectively. The partial substitution of FA and BT with OPC declined the formation of CH in the hydration products. This could be attributed to the consumption of CH during the pozzolanic reaction and dilution effect.

### 4.6. XRD Analysis 

**Figure 10:**
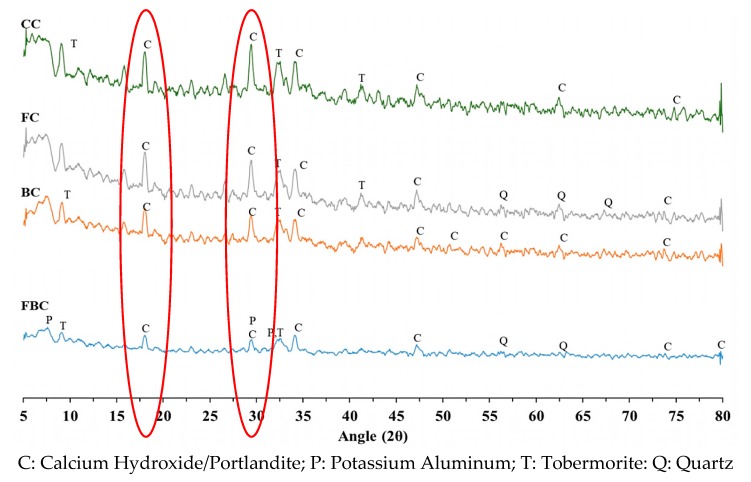
XRD pattern for mass concrete samples containing BT and FA.

The mineralogical compositions of BT and FA samples were analyzed by X-ray diffraction analyses at 28 days of curing. The peaks of calcium hydroxide (CH) and tobermorite, which is a crystalline form of calcium silicate hydrate mineral (C–S–H) [30], were analyzed at 18°, 28.5°, 74° (2ϴ), and 32.5° (2ϴ), respectively. Figure 10 demonstrates XRD patterns of mix containing BT and FA individually and collectively [18].

In the control mix (CC), strong peaks of CH were observed, indicating that a pure hydration product was obtained from the cement hydration. In addition, lower crests of tobormorite were also seen. The substitution of FA retarded the CH peaks, and peaks indicating quartz were observed at 62.5° and 67.8°, which indicated the presence of crystalline silica in FA samples. The reduction in CH concentration is attributed to a decrease in the amount of cement and the pozzolanic reaction of FA. This caused the consumption of free CH available due to the presence of active silica to form a supplementary hydration product. The addition of BT further decreased the intensity of CH peaks. This can be attributed to two reasons. Firstly, a reduction in CH is due to the product of the clinker dilution effect that can be initiated by substituting the portion of cement with an equal quantity of BT. Secondly, BT forms a secondary calcium silicate gel by reacting with the hydrated lime OPC [22]. The combination of 15% FA and 15% BT displayed higher CH consumption. The significant reduction in the peaks of CH in the FBC mix decreased to less than 55%, compared to the peak strength in CC. This showed that the presence of BT and FA improved the hydration products through pozzolanic activity by transforming crystalline phase into amorphous phase.

### 4.7. Morphological Investigation

**Figure 11:**
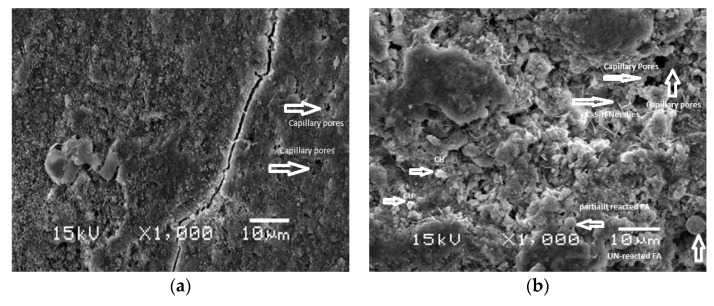

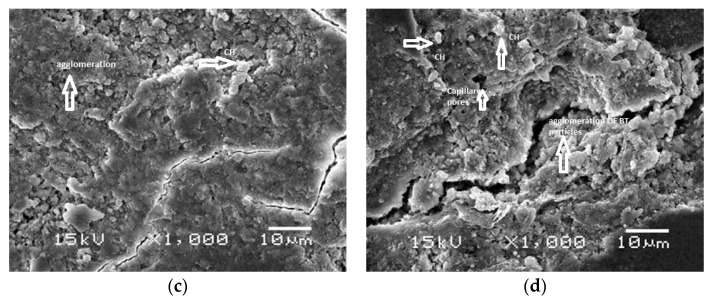
Microstructure of concrete specimens containing FA and BT: (**a**) CC; (**b**) FC; (**c**) BC; (**d**) FBC.

The morphological investigation of samples was analyzed through the scanning electron microscopic (SEM) technique. The micrographs of CC and concrete containing FA and BT separately and collectively are shown in Figure 11. It can be seen that extensive morphological changes are observed with the substitution of BT and FA in cement paste. The fracture of concrete samples exhibited that the hydration products were chiefly in amorphous and crystalline phases of CH and C–S–H gel. The shape of particles of FA was found to be spherical with a smooth surface. However, BT particles had an irregular shape and rough texture, like cement particles. The finer particles of FA were wrapped up by hydration products to be covered in the cement matrix. However, some of the coarser FA particles remained unreacted. It has been reported that coarser particles of FA produced the formation of more hydration product via the pozzolanic reaction [31]. Mix containing BT showed a uniform microstructure due to its finer particle size. The hydration of BC and FBC consumes CH and utilizes it for the formation of additional C–S–H in paste. The additional CH was seen on the surface FBC and BC mix.

## 5. Conclusion 

This research evaluated the thermal, physico-chemical, microstructural, and mechanical behaviour of mass concrete using BT and FA separately and in combination. Four different types of mix ratios were prepared with the substitution of 30% BT, 30% FA, a combination of 15%BT-15%FA, and control mix. The data for binary and ternary blended mixes of BT and FA were obtained using an experimental investigation. The analysis of results was done by comparative analysis, in which the mix-of different proportions was compared with control mix. The following conclusions have been made from the analysis of the results:

(1) The development rate of compressive strength and UPV values in mass concrete samples were significantly improved with the substitution of BT and FA compared to control concrete.
(2) Blends of BT and FA significantly retarded the rise in temperature in mass concrete specimens. Incorporation of FA presented a lower temperature among all the mixes, whereas BT was also found to be more effective to control the rise in temperature.
(3) The XRD analysis revealed that a binary and ternary blend of BT and FA presented a significant reduction in CH peak intensity. This was due the reduction in the cement content and the pozzolanic reaction. The free CH was consumed by silica, which produced additional hydration product in the mix.
(4) DTA/TGA investigations showed a significant reduction in CH amount in BT and FA samples due to the OPC dilution factor and pozzolanic reaction. The reduction in the CH amount of FC, BC, and FBC was calculated as 33%, 40%, and 28%, respectively.
(5) Microstructural analysis through SEM offered a more refined pore-structure with the substitution of BT. The SEM analysis further showed that hydration in the BC and FBC mix consumed CH in the formation of the supplementary hydration product of C–S–H.

## Figures and Tables

**Table 1:** Chemical composition of binders.

Oxides Composition	SiO_2_	Fe_2_O_3_	Al_2_O_3_	CaO	Na_2_O	MgO	MnO	K_2_O	P_3_O_5_	TiO_2_	LOI
	(%)										
Cement	22.19	1.14	6.56	65	0.13	1.07	0.02	1.3	0.06	0.14	2.45
Bentonite	56.13	3.23	21.2	3.16	4.14	4.32	0.05	1.01	0.12	0.55	6.11
Fly ash	48.22	1.19	29.27	9.6	1.84	2.56	0.01	1.14	0.18	0.26	5.78

**Table 2:** Proportions of ordinary Portland cement (OPC), fly ash (FA), and bentonite (BT) in mass concrete specimens.

Mix ID	Mix Description	OPC (%)	FA (%)	BT (%)
CC	Control concrete	100	0	0
FC	Fly ash concrete	70	30	0
BC	Bentonite concrete	70	0	30
FBC	Fly ash and bentonite concrete	70	15	15

**Table 3:** Mix Design.

Mix ID	Kg for 1 m^3^ Concrete						Slump
	Cement	BT	FA	Sand	Gravel	Water	mm
CC	390	0	0	688	1123	203	55
FC	273	0	117	688	1123	203	72
BC	273	117	0	688	1123	203	28
FBC	273	58.5	58.5	688	1123	203	61

**Table 4:** Quality of concrete corresponding to UPV values [26].

**Grading**	Good concrete	Moderate concrete	Poor concrete
**Pulse Velocity**	>4 km s^−1^	4 km s^−1^ > UPV > 3 km s^−1^	<3 km s^−^^1^

**Table 5:** Strength and UPV values of inner and outer cores of all four groups.

Mix	Compressive Strength (Mpa)			UPV (km/s)		
	Outer	Inner	Cylinder	Outer	Inner	Cylinder
CC	21.86	18.06	21.67	4.021	3.991	4.066
FC	25.29	22.86	20.18	4.415	3.975	4.566
BC	19.48	18.97	19.70	4.078	3.881	4.501
FBC	22.33	21.96	20.52	3.928	3.776	4.296
